# Strengthening Research Capacity—TDR's Evolving Experience in Low- and Middle-Income Countries

**DOI:** 10.1371/journal.pntd.0003380

**Published:** 2015-01-08

**Authors:** Olumide A. T. Ogundahunsi, Mahnaz Vahedi, Edward M. Kamau, Garry Aslanyan, Robert F. Terry, Fabio Zicker, Pascal Launois

**Affiliations:** 1 Special Programme on Research and Training in Tropical Diseases (TDR), a co-sponsored programme of UNICEF/UNDP/World Bank/WHO, based at the World Health Organization, Geneva, Switzerland; 2 Center for Technological Development in Health (CDTS), FIOCRUZ, Rio de Janeiro, Brazil; Lindsley F. Kimball Research Institute, New York Blood Center, United States of America

## Introduction

In the 1970s, very few international programmes provided support to strengthen tropical disease research capacity and most research for the diseases prevalent in low- and middle-income countries (LMICs) was done by scientists and institutions in advanced industrialised countries. Soon after inception in 1974, TDR established a research capacity strengthening (RCS) programme with a goal to train individuals and strengthen research capacity in disease-endemic countries so that they can find and implement appropriate solutions to their health problems [1], [2]. At that time, very little research addressed the burden of these diseases. For most of its existence, up to a third of TDR's total resources were earmarked for strengthening research capacity in LMICs. In the past 20 years, other charities, foundations, health research councils, and development agencies have begun their own capacity strengthening programmes, so today, the concept is well accepted, although the means to achieve the end vary [3]–[5]. This paper presents a broad description from the TDR secretariat's perspective on evolving approaches used to promote research capacity strengthening in LMICs. The paper is part of a special series commemorating TDR's 40-year anniversary.

TDR has an intertwined approach: training support for individuals and collaborative research programmes for institutions [1], [2]. Research training requires adequate research facilities, which may need strengthening. Similarly, strengthening an institution so that it can fully participate in a research partnership often calls for supporting training facilities and staff. The specific needs and priorities that are funded by TDR have been identified by a capacity building steering committee and approved by the TDR Scientific and Technical Advisory Committee (STAC), which comprises 15 to 18 experts in a wide range of scientific disciplines who peer review the programme's scientific and technical activities.

TDR's placement within the United Nations system provides close collaboration with country offices of not only the World Health Organization but also of other co-sponsoring agencies UNICEF and UNDP, and with the World Bank. As a consequence, those who are supported by TDR often work closely with disease control programmes as well as other international organizations.

Regular reviews of TDR's research capacity strengthening programmes have helped reorient the strategy as needed, shifting focus from institutional strengthening in the 1980s to human resources strengthening in the 1990s [1], as well as identifying the need to move to a more demand-driven model of national health research systems [4]. Over the years, TDR has continued to support multidisciplinary research, particularly to bring social science research and biomedical research together through different mechanisms [6], and has reinforced this effort through training in implementation research [7] and operations research [8].

## Institutional Strengthening

TDR began by focusing on institutions. Early programmes were non-competitive, long-term grants designed to help institutions acquire or upgrade their existing research capacities. A decade later, 89 institutions had received support, and with this expanded viable scientific community in low- and middle-income countries, the grants evolved into increasingly competitive formats [2]. By 1987, grants required a scientific project of genuine merit, related when possible to local disease-control needs. This philosophy was extended with the creation of programme-based grants that focused on specific areas, such as biotechnology initiatives, social science aspects of disease, or a particular disease, and were judged on a fully competitive basis. Additional innovations were that the proposal was required to link directly to disease control, and funds were allocated to train junior scientists.

In 1988, TDR introduced the partnership grants scheme, jointly funded with the Rockefeller Foundation [2], [9]. These grants encouraged research collaboration between LMIC institutions and another advanced or more experienced institution (often in Europe, North America, or Australia). They provided up to 5 years of support to an institution or research group to develop a research infrastructure and environment by improving training facilities, scientific expertise in biomedical and social sciences, and information and communications systems, while fostering opportunities for scientific collaboration. The grants also included a staff development component to support postgraduate students within institutions.

This competitive approach invariably gave more established institutions and past recipients of TDR long-term grants in middle-income countries an inadvertent advantage over low-income countries with limited research capacity. However, countries with limited research capacity consider TDR support as an essential catalyst for capacity building [10]. As a result, at different times in TDR's history there have been competitive grants exclusively targeting low-income countries to help them increase their capacities.

From 1974 to 2010, TDR awarded grants to over 490 institutions in 45 countries (source: TDR Information Management System). The impact of this long-term support can be seen in a number of areas. For example, the partnership model was used for the successful grants programme of the Multilateral Initiative on Malaria (MIM), which paired institutions from the “North” and the “South”, along with individual training. Most of those principal investigators are now leading health research academic and public health programmes, as well as playing key national and international roles in research governance [11]–[13]. Their research has provided evidence for change in national malaria control policies, such as the introduction of long-lasting insecticide-treated bednets and information on insecticide resistance patterns in malaria vectors, and facilitated the use of community-based management of malaria to reduce malaria associated morbidity and mortality in endemic communities.

Specific institutions have actually been created with TDR support, such as the Malaria Research and Training Centre in Bamako, Mali, which evolved out of a university department in 1988. TDR's support was a catalyst for funding from other countries and sources, resulting in a core national resource (see Box 1) that now conducts research in a number of diseases.

**Box 1. Malaria Research and Training Centre, Bamako, Mali**

**Seed funding helps attract additional support and international collaborations**

A TDR–Rockefeller Foundation Partnership grant (1988–1993) on modification of the *Anopheles gambiae* malaria vector population was the seed for the establishment of a new research and training centre on tropical diseases in Bamako, Mali. Dr. Yeya Touré, whose research sparked this support, was named the Malaria Research and Training Centre's first director. He worked in collaboration with the US National Institutes of Health (NIH) Laboratory of Parasitic Diseases as well as the Institute of Parasitology of the University of Rome “La Sapienza.”

The centre began with studies on vector biology, ecology, and genetics, and quickly expanded to include malaria parasitology, epidemiology, social sciences, molecular biology of vectors and parasites, immunology, geographical information systems/remote sensing, transmission-blocking, clinical research, and vaccine research with the arrival of Dr. Ogobara Doumbo.

A second 4-year TDR grant supported extensive research and training activities on malaria and schistosomiasis. This was complemented by significant funding from US Agency for International Development (USAID) and NIH to build over 20 new and fully equipped laboratories and a guest house and to provide field vehicles, intranet local area network, and internet connectivity (very small aperture terminal [VSAT]). The centre also attracted other funding from French and Italian cooperation, the International Atomic Energy Agency (IAEA), and the European Commission, to name a few of the many sources of support.

Dr. Touré left the centre in 2001 to the leadership of Ogobara Doumbo and Sékou Traoré and came to TDR to manage an international malaria research portfolio. By then, the number of researchers had increased from four to more than 30 and the research scope had expanded to lymphatic filariasis, leishmaniasis, tuberculosis, HIV-AIDS, and other diseases, in support of national disease-control programs. In 2004, the Malaria Research and Training Center (MRTC)and the HIV/AIDS programs became part of the Mali International Center for Excellence in Research (ICER), which today is a leading national institution managed by ICER staff on behalf of the Faculty of Medicine and Pharmacy and NIH.

TDR also helped support networks and centres of excellence so that groups could learn from each other [14]. The African Network for Drugs and Diagnostics Innovation (ANDI) was launched by TDR in 2008 [15]. This came out of an analysis of research partnerships that showed that the majority of partnerships were between African institutions and institutions in Europe and the United States, and that there were opportunities to increase networking among African institutions and, eventually, also increase research and development (R&D) production and manufacturing on the African continent [16]. With an initial grant from the European Commission, Pan-African centres of excellence were identified and promoted as a first step toward this goal, a Board and secretariat were established, and the programme is now being transitioned to the United Nations Office for Project Services (UNOPS) as an independent entity.

More recently, TDR Regional Training Centres have been helping institutions become hubs for skill acquisition in the WHO regions, such as in Cali, Colombia (see Box 2). These centres provide courses in organizing, managing, and conducting health research, which help researchers to improve their research quality and competitiveness in getting grants. There are currently four centres in operation—Centro Internacional de Entrenamiento e Investigaciones Médicas in Colombia for the Americas, Gadjah Mada University in Indonesia for the Southeast Asian region, Astana Medical School in Kazakhstan for the European region, and the Research Institute of Tropical Medicine in the Philippines for the Western Pacific region. Two more centres will be established, one in Africa and the other in the Eastern Mediterranean region.

**Box 2. CIDIEM, Cali, Colombia**

**From one course and one centre to a Latin American network**

It started in Cali, Colombia in 2007 with project management. TDR had developed a course—Effective Project Planning and Evaluation (EPPE)—to train scientific teams in how to help plan and evaluate their work, and also provided a train-the-trainer programme and manuals so that the course could be used within academic institutions and research groups. The Centro Internacional de Entrenamiento e Investigaciones Médicas (CIDEIM) was interested in using this programme, and thus began a fruitful collaboration that has spread throughout the Latin American region.

TDR provided funding to implement the course in English at CIDEIM, where it was enthusiastically taken up and eventually translated into a Spanish, online version. In 2010, CIDEIM was named the first TDR Regional Training Centre, which provided funding for trainers to go to neighbouring countries to give the course and expand the number of trainers. Institutions in Ecuador, Honduras, Guatemala, Cuba, and Jamaica have implemented the EPPE course so far. Course participants are engaged as a team, with some being supported by their institutions or by the Pan American Health Organization. The course is also being incorporated into curricula for undergraduate and postgraduate programmes.

In just 4 years, CIDEIM has established an expanded network that is building research skills and support. Additional courses have been developed in statistics for biomedical research, good clinical practice, data analysis planning, and budget planning. A new course on the use of information and communication technology for health research is currently being designed and developed with the support of a TDR impact grant.

## Support for Individuals

A variety of individual training grants have been provided over the years, often integrated with institutional support. The cornerstone of individual capacity development support has been the research training grant awarded to young scientists to pursue studies leading to a postgraduate degree (MSc, PhD) or acquisition of specialised skills through short-term courses and postdoctoral training. During the period from 1975 to 1996, a total of 1,438 postgraduate training grants were awarded, with one-third of these going to candidates from least developed countries [17]. During the first 10 years, the average total cost of PhD training in the United Kingdom and the US was established to be about US$ 100,000. The high cost, as well as the challenges faced by young scientists during and after a prolonged stay outside their home environment, informed a shift to the “sandwich” PhD programme in the mid-1980s [18]. This programme entailed an initial period of course work at the academic institution abroad followed by a period of research conducted in the grantee's home country, with a third and final period to write and defend the thesis at the academic institution abroad. The grant was designed to combine excellence with relevance while facilitating reintegration of the graduate professional to their home institution [18]. During the third decade (between 1997 and 2010), 356 postgraduate training grants were awarded. More than 85% of past grantees of this programme returned to their home countries and remained active in their field of study, attaining senior or leadership positions in government or academic institutions [10]. In 2011, TDR went through a major restructuring and very few grants were awarded across the programme. However after an interregnum of 3 years, 22 new postgraduate research training grants and 9 postdoctoral fellowships were awarded in 2014.

Recognizing the challenges encountered during the early stages of a scientific career such as re-establishment at the home institution, TDR developed the re-entry grants scheme. Re-entry grants are comprised of research-based 2-year support intended to facilitate the career development of young scientists returning to their home institutions within 12 to 24 months of completing a graduate degree [2]. The grants were awarded based on scientific merit and have been instrumental in establishing new research groups and providing opportunities for independent research [10].

Other modalities of individual support included: grants for visiting scientists to participate in research and training activities in another institution, postgraduate research grants to support basic and applied research outside the training institution, and the career development grants, which included funding for research and international travel to assist outstanding investigators to remain in a research environment and establish a continuing career. These grants have facilitated international experience and opportunities to learn from other research environments.

## Sustaining Research Capacity

To further strengthen and sustain research capacity in LMICs, TDR is addressing three critical issues: 1. career path recognition; 2. institutional leadership; and 3. capacity relevant to national research priorities.

First, career path recognition is a major challenge in LMICs. Usually postdoctoral professionals have limited time to conduct research as they are in high demand by the universities for full-time teaching. Consequently, only a few can devote quality time to conduct research and publish to advance their careers and gain recognition within the scientific community. A productive scientific career is known to increase the chances of obtaining research funds, while a heavy teaching load limits the opportunity to learn grantsmanship and improve scientific productivity. Although a few grant schemes, such as the re-entry, have helped the scientific career of post-docs in academic institutions, until recently, postdoctoral research positions with no teaching responsibilities were the exception in many LMIC universities. The importance of linking teaching to research is now beginning to receive more attention. The Conseil Africain et Malgache pour l'Enseignement Supérieur/African and Malagasy Council for Higher Education (CAMES), an intergovernmental institution in Francophone Africa that was established to harmonise the operations of emerging African universities and integrate higher education systems, now promotes research among its members across 17 African countries. To this end, in 2013, CAMES organised its first scientific conference focusing on research.

Another development is the Postdoctoral Fellowship Program at the Noguchi Memorial Institute for Medical Research (NMIMR), University of Ghana. Recently established with support from the Bill & Melinda Gates Foundation, the program seeks to “train young African scientists and equip them to compete effectively for international funding for research work in Africa, offer them international exposure, and facilitate their networking and collaboration with other institutions” [19]. More commitment and recognition of the value of research as well as willingness to change is needed from the institutions themselves to strengthen and sustain research capacity in LMICs [20], [21].

The second and related challenge is the development of institutional leadership. Leadership is recognised as an important element for institutional scientific productivity, while poor management poses significant risk to sustained research capacity [22], [23]. A 1991 review by STAC [2] found that the academic qualifications of a principal investigator is not a good indicator of the success or failure of a research strengthening programme and that success was, in fact, associated with capable, committed leadership and stable, long-term linkages with other institutions. As a result, STAC recommend that TDR identify and support talented individuals as potential leaders of institutions and managers of research. Identifying and supporting the individuals with the requisite managerial skills remains a challenge. Training programmes have been developed by some organizations to help scientists build relevant skills early in their careers [24]. The development of leadership requires consistent, long-term investment, as well as mentorship in a wide range of skill sets, such as priority setting, strategic planning, conflict resolution, negotiation (including intellectual property), risk management, budgeting, human resource management, and team building. At the institutional level, a research office to manage, organise, and improve the competitive performance of research, including rising external funding and reporting, are *sine qua non* for growth and sustainable research capacity. Some of these, as well other factors, have been previously identified [22], [23].

TDR offered leadership training grants in 2007 to complement grantees' biomedical and applied research skills with those in research management, leadership, priority setting, and other areas of relevance. Grants were awarded on a competitive basis to researchers recommended by the home institution. Implementation was flexible and included a visit to TDR's offices in Geneva during the World Health Assembly. The aim was to provide exposure to global public health governance and dynamics while giving grantees the opportunity to meet with health policy-makers. Ten leadership training grants were awarded. Unfortunately, the scheme failed to produce the expected impact on the host institution's research capacity. Once the candidate had been recommended, TDR was unable to maintain a strong link with the leadership of the institution during the 3-year period of the grant. Although the concept was endorsed by the advisory committee, it proved difficult to execute, and it was not possible to evaluate contribution of the grant to institutional capacity development. The scheme was discontinued in 2010.

In an effort to develop research management skills, TDR is currently disseminating a project-planning and evaluation course through its regional training centres. An online continuing professional development platform has also been piloted with support from the Bill & Melinda Gates Foundation and the Global Health platform [25]. The Web-based platform was designed to help promote ongoing professional development that could lead to leadership positions. It provides a mechanism to capture and record core competencies, qualifications, and training in order to accrue points and work up through five membership tiers, with recognition of the individual's productivity. The platform was renamed the Professional Membership Scheme (PMS) and was subsequently opened to all scientists in 2012. Within a 12-month period, there were 292 registered members and more than 5,000 individual visitors.

Other attempts to foster research capacity and leadership include the South-South initiative for Infectious diseases of poverty (SSI), introduced by TDR's defunct pathogenesis and applied genomic committee. Such collaborations were meant to harness the diverse research and training capability in disease-endemic countries and promote interaction and research collaboration between investigators across Africa, Latin America, and Asia. It is expected that in the long term, sustainable structures, such as national research councils, will adopt this approach and broaden their reach to benefit other LMICs, as is the case with Brazil [26]. The dynamic and sometimes unanticipated global health challenges and changes in disease epidemiology are a compelling case for such collaboration.

A third challenge is support for research capacity that is relevant to national priorities. Given the pressing disease-control issues in many countries, research should be responsive to public health needs and be coordinated closely with national authorities to facilitate uptake of results. Scientists need to be more involved in disseminating their research results to different stakeholders. The concept of publishing in a peer-reviewed journal should not be reinforced as an end in itself. Translating research results into policy recommendations, concrete interventions, or new tools have been identified as major weaknesses of research capacity strengthening programmes [27]–[29]. It can be argued that in public health, the metric of productivity should be contribution of research to improved policies and practices through the application of the knowledge and evidence generated.

Two new initiatives in TDR are now focussing on research responding to public health needs in LMICs. SORT IT [8], a joint programme of TDR, the International Union Against Tuberculosis and Lung Disease, and Médecins Sans Frontières, helps public health programmes in LMICs to identify and overcome their operational challenges to improve performance and impact on disease burden. The Implementation Research Toolkit [7] was developed to help multidisciplinary research teams to address implementation bottlenecks and to identify and disseminate appropriate solutions to stakeholders.

The fragmented research and capacity building funding related to the large number of agencies and actors has contributed to some extent to selective funding to some research groups and institutions, while others that could have a more direct and immediate impact in the countries have been neglected.

To address this disparity, TDR helped initiate the ESSENCE on Health Research initiative [30] in 2009. ESSENCE is an international collaboration between research funders, development agencies, philanthropists, and multilateral initiatives. It includes major funders such as The Wellcome Trust, the Swedish International Development Cooperation Agency, Canada's International Development Research Centre, and the US National Institutes of Health/Fogarty International Centre. With the secretariat hosted in TDR, ESSENCE provides a mechanism for collaboration between funders as well as between funders and recipient countries to coordinate, harmonise, and align funding and research activities with countries' health agendas. ESSENCE has three main areas of focus: (1) policy dialogue; (2) areas for harmonization; and (3) country pilots. A key achievement has been the agreement by members to jointly develop and produce a series of good practice documents. In the last 4 years, these documents have incorporated current best knowledge and practice on health research and development issues in such areas as planning, monitoring, and evaluation of capacity strengthening in health research, research costing in LMICs, and principles of research capacity strengthening [31]–[32].

TDR has successfully accomplished its mandate strengthening research capacity through three generations of scientists in LMICs. Although there has not been consensus on the best way to assess the impact of TDR support, the current strategy of planning by results has allowed the programme to establish targets and milestones, which are reported routinely to the STAC [33]. Competing priorities in terms of scope and emerging opportunities are prioritised in consultation with advisory committees.

In summary, strengthening and sustaining research capacity in LMICs involves many interrelated factors at the levels of the individual, the institution, and the leadership (see Box 3). TDR recognises the need for increased support for the individual during the postdoctoral period, the development of leadership skills, and broader linkages and networks for researchers. These gaps are being addressed through a flexible impact grants scheme, postgraduate and postdoctoral training schemes, career development fellowships in pharmaceutical companies, and regional training centres. There are also efforts to address the ongoing issue of unequal gender representation among researchers in LMICs (as elsewhere). A Web-based alumni network for current and past grantees is under development to facilitate mentoring, further analyse what works best, and connect researchers to new resources, independent of whether they have current TDR grant support or not. As the programme goes forward, TDR's research capacity strengthening support will focus on developing the skills and evidence needed to directly address health problems in LMICs, as well as output-oriented training targeted directly at those working in public health interventions, to increase the generation and uptake of evidence to strengthen implementation [34].

**Box 3. Some Enablers and Barriers to Sustainable Research Capacity**

**Enablers**

1. Capable and committed scientific leadership
2. Clear institutional vision and contextual relevance
3. Continuity of funding for research
4. Ability to attract a core of dedicated young scientists and providing them with independent research funding
5. Adequate and appropriate infrastructure for research (buildings, premises, procurement and financial management systems)
6. Adequate equipment and supplies, including communication facilities and access to scientific literature
7. Partnerships, networks, and research collaboration (regional and global)
8. Career paths for research staff with adequate rewards for productivity
9. Adequate and responsible mentoring

**Barriers**

1. Weak scientific leadership
2. Diversion of researchers to other, nonscientific tasks
3. Strong external (usually political) influences on the running of the institution
4. Strong adverse local sociopolitical climate, creating frustration among the scientists
5. Poor remuneration, compelling the scientists to either seek other sources of remuneration to augment their income or leaving for other institutions
6. Inappropriate service conditions, resulting in departure of scientists
7. Poor internal quality control systems

Adapted from [18]

## References

[1] Ridley RG, Fletcher ER (2008) Making a difference: 30 years of TDR. Nat Rev Microbiol 6(5): 401–407. 18414503 10.1038/nrmicro1899

[2] World Health Organization Tropical Disease Research (1995) Twelfth Programme Report of the UNDP/World Bank/WHO Special Programme for Research and Training in Tropical Diseases. Progress 1975–94, Highlights 1993–4.

[3] Lansang MA, Dennis R (2004) Building capacity in health research in the developing world. Bull World Health Organ 82(10): 764–770. 15643798 PMC2623028

[4] Ghaffar DA, IJsselmuiden C, Zicker F (Eds.). (2008) Changing mindsets: Research capacity strengthening in low-and middle-income countries. Council on Health Research for Development (COHRED). 15p Available: http://www.cohred.org/publications/cohred-publications/joint-publications/changing-mindsets-research-capacity-strengthening-in-low-and-middle-income-countries/.

[5] Daar AS, Whyte SR, Mohamed SA, Ching-Li H, Hoffman SL, et al. (2008) TDR thirty years on: taking stock and envisioning the future for the Special Programme for Research and Training in Tropical Diseases. PLoS Negl Trop Dis, 2(11): e314. 10.1371/journal.pntd.0000314 PMC2582684 19030222

[6] Sommerfeld J, Ramsay A, Pagnoni F, Terry RF, Guth J, Reeder JC (2014) Applied research for better disease prevention and control. PLoS Negl Trop Dis, 10.1371/journal.pntd.0003378 10.1371/journal.pntd.0003378 PMC4287520 25568958

[7] WHO/TDR (2014) Implementation Research Toolkit. Available: http://www.who.int/tdr/publications/topics/ir-toolkit/en/ Accessed 6 November, 2014

[8] WHO/TDR (2013) Structured operational research and training website. Available: http://www.who.int/tdr/capacity/strengthening/sort/en/ Accessed 6 November, 2014.

[9] Lansang MAD, Olveda RO (1994) Institutional linkages: strategic bridges for research capacity strengthening. Acta Trop 57(2): 139–145. 7985548 10.1016/0001-706x(94)90004-3

[10] Minja H, Nsanzabana C, Maure C, Hoffmann A, Rumisha S, et al. (2011) Impact of health research capacity strengthening in low-and middle-income countries: the case of WHO/TDR programmes. PLoS Negl Trop Dis 5(10), e1351. 10.1371/journal.pntd.0001351 PMC3191138 22022630

[11] Nantulya FN, Kengeya-Kayondo JF, Ogundahunsi OAT (2007) Research Themes and Advances in Malaria Research Capacity Made by the Multilateral Initiative on Malaria. Am J Trop Med Hyg 77: 303–313. 18165507

[12] Hotez PJ (2008) Tropical diseases research: thirty years and counting. PLoS Negl Trop Dis 2(11), e329. 10.1371/journal.pntd.0000329 PMC2582687 19030224

[13] Ntoumi F, Priebe G (2010) Africanizing scientific knowledge: the Multilateral Initiative on Malaria as a model? Malar J 9 (Suppl 3), S7. 10.1186/1475-2875-9-S3-S7 PMC3002143 21144087

[14] Zhou XN, Wayling S, Bergquist R (2010) Concepts in Research Capabilities Strengthening: Positive Experiences of Network Approaches by TDR in the People's Republic of China and Eastern Asia. Adv Parasitol 73: 1–19. 20627137 10.1016/S0065-308X(10)73001-3

[15] Nwaka S, Ochem A, Besson D, Ramirez B, Fakorede F, et al (2012) Analysis of pan-African Centres of excellence in health innovation highlights opportunities and challenges for local innovation and financing in the continent. BMC Int Health Hum Rights 12: 11. 22838941 10.1186/1472-698X-12-11 PMC3492037

[16] Nwaka S, Ilunga TB, Da Silva JS, Verde ER, Hackley D, et al. (2010) Developing ANDI: a novel approach to health product R&D in Africa. PLoS Med7(6), e1000293. 10.1371/journal.pmed.1000293 PMC2893959 20613865

[17] United Nations Office of the High Representative for the Least Developed Countries, Landlocked Developing Countries and Small Island Developing States. About LDCs (Least Developed Countries) website. Available: http://unohrlls.org/about-ldcs/ Accessed 11 November 2014.

[18] Nchinda TC (2002) Research capacity strengthening in the South. Soc Sci Med 54(11): 1699–1711. 12113452 10.1016/s0277-9536(01)00338-0

[19] Noguchi Memorial Institute for Medical Research (05 Nov 2014). Call for applications NMIMR postdoctoral training fellowships. Available: http://www.noguchimedres.org/index.php/86-news/286-postdoctoral-training-program-at-noguchi-extension-of-call Accessed 11 Dec 2014

[20] Whitworth JA, Kokwaro G, Kinyanjui S, Snewin VA, Tanner M, et al (2008) Strengthening capacity for health research in Africa. Lancet 372(9649): 1590–1593. 18984193 10.1016/S0140-6736(08)61660-8 PMC2607030

[21] Bland CJ, Ruffin MT (1992) Characteristics of a productive research environment: literature review. Acad Med 67(6): 385–397. 1596337 10.1097/00001888-199206000-00010

[22] White F (2002) Capacity-building for health research in developing countries: a manager's approach. Rev Panam Salud Publica 12(3): 165–172. 12396634 10.1590/s1020-49892002000900004

[23] Wides C, Mertz E, Lindstaedt B, Brown J (2014) Building Leadership among Laboratory-Based and Clinical and Translational Researchers: The University of California, San Francisco Experience. Clin Transl Sci 7(1): 69–73. 24405661 10.1111/cts.12135 PMC4224438

[24] Howard Hughes Medical Institute and Burroughs Wellcome Fund (2006) Making the Right Moves: A Practical Guide to Scientifıc Management for Postdocs and New Faculty. Chevy Chase: Howard Hughes Medical Institute & Research Triangle Park: Burroughs Wellcome Fund. 267 p. Available: http://www.hhmi.org/programs/resources-early-career-scientist-development/making-right-moves: Accessed 31 October 2014.

[25] Global Health Trials Professional membership scheme. Available at: https://globalhealthtrainingcentre.tghn.org/cpd/about/ Accessed 11 November 2014.

[26] Almeida C, Pires de Campos R, Buss P, Ferreira JR, Fonseca LE (2010) Brazil's conception of South-South “structural cooperation” in health. Rev Electron Comun Inf Inov Saude 4: 23–32.

[27] Gilson L, Raphaely N (2008) The terrain of health policy analysis in low and middle income countries: a review of published literature 1994–2007. Health Policy Plan 23(5): 294–307. 18650209 10.1093/heapol/czn019 PMC2515407

[28] IJsselmuiden C, Matlin S (2006) Why health research? Ginebra: Global Forum for Health Research.

[29] Whitworth J, Sewankambo NK, Snewin VA (2010) Improving implementation: building research capacity in maternal, neonatal, and child health in Africa. PLoS Med 7(7), e1000299. 10.1371/journal.pmed.1000299 PMC2897765 20625547

[30] WHO/TDR (2014) ESSENCE on health research website. Available: http://www.who.int/tdr/partnerships/initiatives/essence/en/. Accessed 6 November 2014.

[31] ESSENCE (2011) Planning, monitoring, and evaluation framework for capacity strengthening in health research. Available: http://whqlibdoc.who.int/hq/2011/TDR_essence_11.1_eng.pdf?ua=1. Accessed 6 November 2014.

[32] ESSENCE (2012) Five keys to improving research costing in low- and middle-income countries. Available: http://whqlibdoc.who.int/hq/2012/TDR_ESSENCE_1.12_eng.pdf. Accessed 6 November 2014.

[33] WHO/TDR (2013) Monitor, evaluate, Improve. TDR Results: 2013 Report. Available: http://who.int/tdr/publications/result_report_2013/en/ Accessed 6 November 2014.

[34] Reeder JC (2014) TDR:(Back) Making a Difference. PLoS Negl Trop Dis 8(1), e2531. 10.1371/journal.pntd.0002531 PMC3888447 24421908

